# Structure and interactions of the archaeal motility repression module ArnA–ArnB that modulates archaellum gene expression in *Sulfolobus acidocaldarius*

**DOI:** 10.1074/jbc.RA119.007709

**Published:** 2019-03-22

**Authors:** Lena Hoffmann, Katrin Anders, Lisa F. Bischof, Xing Ye, Julia Reimann, Sunia Khadouma, Trong K. Pham, Chris van der Does, Phillip C. Wright, Lars-Oliver Essen, Sonja-Verena Albers

**Affiliations:** From the ‡ Institute for Biology II, Molecular Biology of Archaea and; the ¶ Spemann Graduate School of Biology and Medicine, University of Freiburg, 79104 Freiburg, Germany,; the § Philipps University, Department of Chemistry, 35032 Marburg, Germany,; the ‡‡ LOEWE Center for Synthetic Microbiology, 35043 Marburg, Germany,; the ‖ ChELSI Institute, Department of Chemical and Biological Engineering, University of Sheffield, Sheffield S1 3JD, United Kingdom, and; the ** Faculty of Science, Agriculture and Engineering, Newcastle University, Newcastle upon Tyne NE1 7RU, United Kingdom

**Keywords:** Archaea, cell motility, protein phosphorylation, signal transduction, transcription regulation

## Abstract

Phosphorylation-dependent interactions play crucial regulatory roles in all domains of life. Forkhead-associated (FHA) and von Willebrand type A (vWA) domains are involved in several phosphorylation-dependent processes of multiprotein complex assemblies. Although well-studied in eukaryotes and bacteria, the structural and functional contexts of these domains are not yet understood in Archaea. Here, we report the structural base for such an interacting pair of FHA and vWA domain-containing proteins, ArnA and ArnB, in the thermoacidophilic archaeon *Sulfolobus acidocaldarius*, where they act synergistically and negatively modulate motility. The structure of the FHA domain of ArnA at 1.75 Å resolution revealed that it belongs to the subclass of FHA domains, which recognizes double-pSer/pThr motifs. We also solved the 1.5 Å resolution crystal structure of the ArnB paralog vWA2, disclosing a complex topology comprising the vWA domain, a β-sandwich fold, and a C-terminal helix bundle. We further show that ArnA binds to the C terminus of ArnB, which harbors all the phosphorylation sites identified to date and is important for the function of ArnB in archaellum regulation. We also observed that expression levels of the archaellum components in response to changes in nutrient conditions are independent of changes in ArnA and ArnB levels and that a strong interaction between ArnA and ArnB observed during growth on rich medium sequentially diminishes after nutrient limitation. In summary, our findings unravel the structural features in ArnA and ArnB important for their interaction and functional archaellum expression and reveal how nutrient conditions affect this interaction.

## Introduction

Stress response that yields adaptation to changing environmental conditions is one of the most important prerequisites to ensure survival in all living organisms. A vast amount of modules has evolved to receive, process, and transfer these signals within the cell. A well-known key element of cellular signal transduction is phosphorylation, which at the same time is one of the most important posttranslational modifications in all three domains of life (1). In prokaryotes sensor kinases receive environmental signals and transmit them to receivers within the cell, which mostly regulate gene expression. A variety of protein families exists, which specifically recognize and bind phosphorylated side chains in target proteins. One representative is the family of proteins containing forkhead-associated (FHA)^4^ domains, which have been intensely studied since their identification in 1995 (2). FHA domains are ubiquitously found and involved in a variety of cellular processes. For example, FHA domains are part of the eukaryotic DNA damage-response, DNA-repair, and DNA-replication systems (3). This domain type is often part of larger kinases, *e.g.* in Rad53 of *Saccharomyces cerevisiae*, where it is involved in the signaling cascade initiated after DNA damage by interaction with other proteins (4). In bacteria, FHA domain–containing proteins are involved in a variety of processes such as amino acid production, sporulation, or resistance to antimicrobial substances. In the latter, either they are part of a protein that directly interacts with DNA and subsequently binds phosphorylated proteins *via* its FHA domain, or they bind to phosphorylated proteins (5–7). The overall structure of the FHA domain is similar in both eukaryotes and bacteria: FHA domains comprise a fold consisting of two β-sheets that are connected *via* loops of variable length to form a twisted β-sandwich. These loops are the main interaction sites with other proteins because they harbor the important pThr-binding motifs (8). Interestingly, this motif selectively binds to pThr and fails mostly to recognize pSer (9). In contrast to eukaryotes and bacteria, where the FHA domain is widely distributed and most genomes encode several proteins with these domains, most archaeal genomes analyzed so far lack proteins containing a FHA domain (10). Only two proteins with a FHA domain have been studied in Archaea to date, both of which belong to a protein family found in several archaea of the class of Thermoprotei. Wang *et al.* (11) modeled the protein St0829 from *Sulfolobus tokodaii* and showed that it possesses a FHA domain with conserved Asn, Ser, and Arg residues in the β-strand–connecting loops. St0829 interacts with a Ser/Thr kinase of *S. tokodaii* and might function as a transcriptional regulator (12). ArnA, an FHA domain containing protein from *Sulfolobus acidocaldarius* was shown to strongly interact with ArnB, a gene product that contains a von Willebrand type A domain (13). von Willebrand type A domains represent a universal scaffold for interaction (14). Initially, this domain was identified in the von Willebrand factor, a blood glycoprotein that sequesters the blood-clotting factor VIII and is involved in platelet adhesion (15). Although von Willebrand factor can only be found in the blood of vertebrates, its A domain (vWA) is found in all three domains of life (16). Structurally, the vWA domain consists of parallel and antiparallel β-strands forming a β-sheet, which is sandwiched by seven α-helices. Like 46% of all domains of the vWA family, it contains a metal ion–dependent adhesion site (MIDAS) that has the consensus D*X*S*X*S and coordinates Mg^2+^-ions together with a further Asp and a Thr residue located elsewhere in the domain. Mg^2+^ binding is crucial for interaction with other proteins as an Ala substitution of the Asp in the D*X*S*X*S motif in the A domain of the integrin CR3 abolishes metal and ligand binding (17, 18). Co-crystal structures of the vWA domain containing α6β1 integrin with laminin-511 show that the interaction occurs via the MIDAS domain in α6β1 integrin and a glutamate residue (Glu-229) in the γ-tail of maninin 511 (19). A conserved glutamate (Glu-78) of WDR12, the mammalian ribosome assembly factor, was also essential for interaction with the MIDAS site of the vWA containing large dynein-like protein midasin (20), suggesting that coordination of the metal in the MIDAS site by a glutamate residue derived from the interaction partner might be conserved in other interactions involving a MIDAS site. Many archaeal species encode at least one vWA domain–containing protein, but no crystal structure has been yet reported.

Here we set out to characterize the structures of, the interactions between, and the biological role of the FHA domain–containing protein ArnA and the vWA domain–containing protein ArnB of *S. acidocaldarius*. ArnA and ArnB regulate gene expression of the archaellum, the motility structure of archaea (13). The archaellum of *S. acidocaldarius* consists of seven proteins (FlaB, FlaX, FlaG, FlaF, FlaH, FlaI, and FlaJ) that are encoded in an operon transcribed from two promoters. Expression of the archaellum is regulated by a complex network of positive and negative regulators (21). Especially the promoter upstream of the gene encoding the archaellum filament protein FlaB is strongly induced during nutrient limitation (22, 23). Deletion of *arnA* and *arnB* caused no strong effects during growth in nutrient-rich conditions, but under nutrient-depleted conditions resulting in higher levels of FlaB and FlaX, more archaella on the surface and hypermotility (13). Phosphorylated ArnA and ArnB have been detected in *S. acidocaldarius*, and it was hypothesized that the interaction between the FHA domain containing ArnA and the vWA domain containing ArnB depends on the phosphorylation state of either of them (13, 24).

Here, we study the interactions between ArnA and ArnB under different conditions and solve the crystal structures of ArnA and vWA2, a close homolog of ArnB, which are the first crystal structures of archaeal FHA and vWA domain–containing proteins. We show that the C-terminal four helix bundle of ArnB is the main site of phosphorylation of ArnB, and deletion of this domain results in a strong reduction of the interaction with ArnA and reduced motility. To our knowledge, this is the first example of interaction between a FHA domain and a vWA domain–containing protein that is phosphorylation-dependent and affects motility.

## Results

### Levels of ArnA and ArnB do not change during starvation

**Figure 1.**
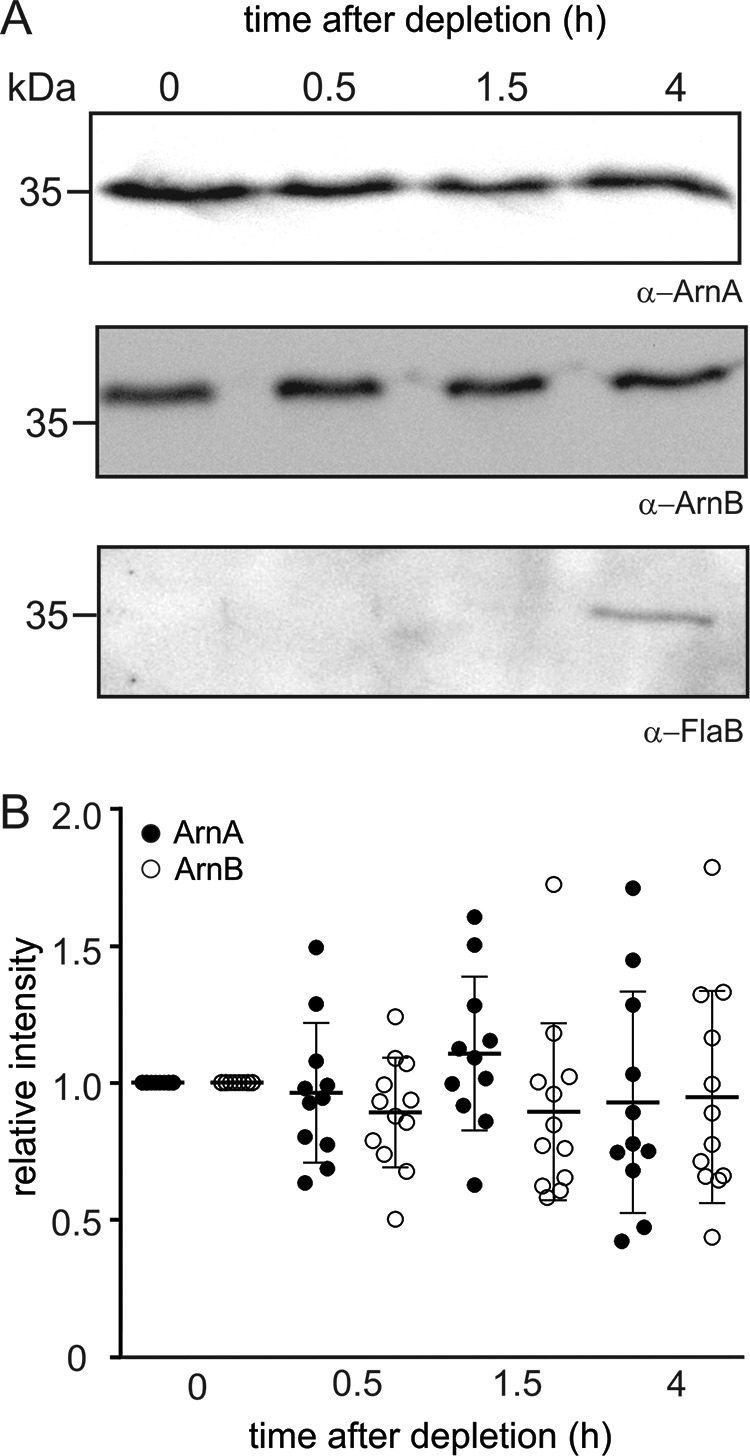
**Protein levels of ArnA, ArnB, and FlaB in *S. acidocaldarius* MW001 (WT) before and after nutrient depletion.** *A*, cells were grown to an *A*_600_ of 0.4 (time point, 0 h) and then shifted to nutrient depleted medium (23, 24). Samples were taken after 0.5, 1.5, and 4 h of nutrient limitation (time points are depicted above the respective lanes), separated on an SDS-PAGE, and analyzed by Western blotting using ArnA (*top row*), ArnB (*middle row*), or FlaB (*bottom row*) specific primary antibodies. The analysis was repeated three times, and a representative Western blot is depicted. *B*, quantification of ArnA and ArnB levels. Levels of three independent experiments and two technical replicates were quantified relative to their intensities at time point 0 h.

In *S. acidocaldarius*, expression of the archaellum is induced upon nutrient limitation (23). Deletions of *arnA* and *arnB* result, under nutrient depletion conditions, in higher *flaB* and *flaX* expression levels, higher numbers of archaella on the surface and hypermotility on plate, demonstrating that ArnA and ArnB play a role in the repression of the expression of archaellum components under these conditions (13). To test whether repression of expression levels of the components of the archaellum occurs via changes in the levels of *arnA* and *arnB* gene products, the protein levels of ArnA, ArnB, and FlaB were determined during exponential growth in rich conditions and under nutrient depletion (Fig. 1). Remarkably, quantification of our Western blotting analysis revealed no significant differences in levels of ArnA and ArnB in the different conditions (Fig. 1*B*), whereas, as previously described, the levels of FlaB were strongly increased under starvation conditions (Fig. 1*A*) (23, 24), thus demonstrating that the expression levels of the archaellum components are not simply regulated via the levels of ArnA and ArnB.

### The interaction between ArnA and ArnB is abolished during nutrient limitation

**Figure 2.**
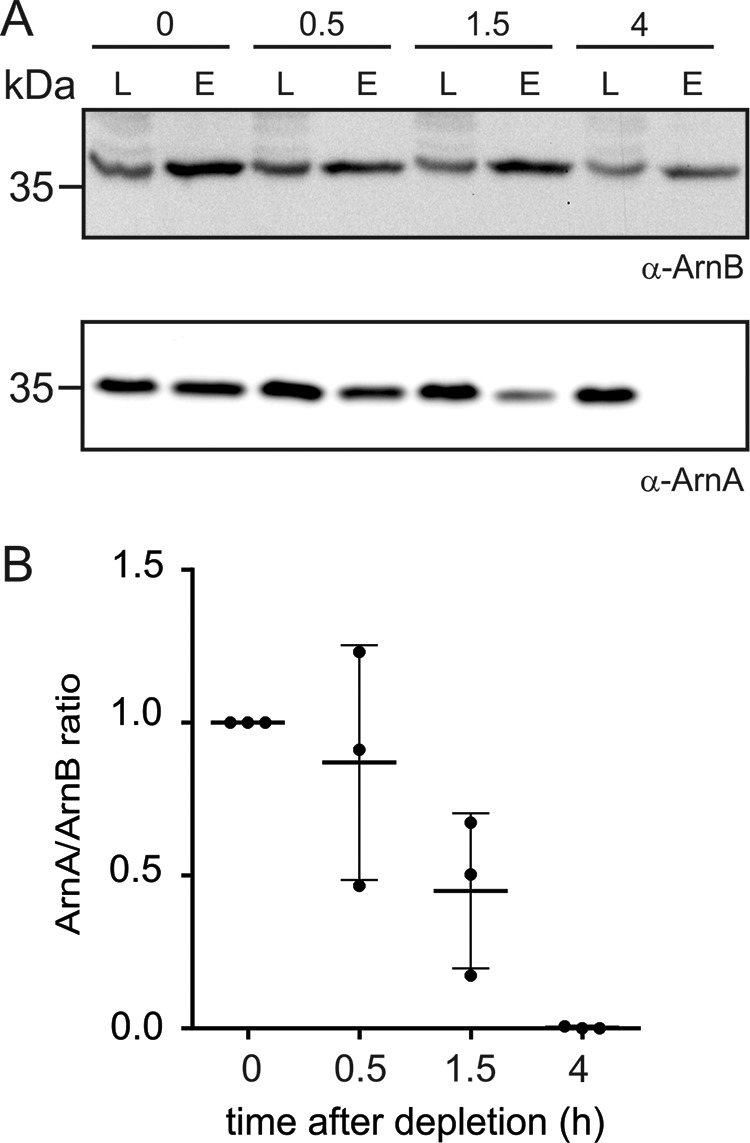
**Interaction of ArnA and ArnB in nutrient-rich and nutrient-depletion conditions.** Pulldown experiments with MW390 (Δ*arnB*::*arnB-Strep*) were performed in exponentially grown cells (*T* = 0 h) and cells growing in nutrient depleted medium for 0.5, 1.5, and 4 h using Strep-Tactin–coated magnetic beads. *A*, ArnB and ArnA were detected in load (*L*) and elution (*E*) fractions using specific primary antibodies. Each pulldown and Western blotting was performed three times with independent replicates, and a representative blot is depicted. *B*, the ratio of ArnA/ArnB found in the elution fractions at the different time points was quantified and averaged for three independent experiments. The ArnA/ArnB ratio at the start of depletion was set to 1.

Because the levels of ArnA and ArnB do not change significantly after nutrient depletion (Fig. 1, *A* and *B*) and ArnA and ArnB have been shown to interact *in vivo* (13), we set out to test whether the interaction between ArnA and ArnB might change upon nutrient depletion. For that, the *arnB* deletion strain was complemented with Strep-tagged *arnB*, and pulldown experiments were performed. The cells were grown to exponential phase in the presence of nutrients and transferred to medium lacking nutrients to induce the expression of the archaellum (Fig. 2*A*) (23, 24). Samples were taken before starvation (0 h) and after 0.5, 1.5, and 4 h of nutrient limitation and subjected to pulldown experiments with streptavidin-coated magnetic beads. As previously observed, in exponentially growing *S. acidocaldarius*, ArnA was found to co-elute with Strep-tagged ArnB (Fig. 2*A*) (13), demonstrating the interaction between the two proteins during nutrient-rich growth. Remarkably, under nutrient-limiting conditions, this interaction got lost (Fig. 2, *A* and *B*). After 0.5 h of starvation, ArnA still co-eluted with ArnB, whereas after 1.5 h the levels of ArnA in the elution fractions were already decreased, and strikingly, after 4 h of starvation ArnA did not co-elute with ArnB anymore (Fig. 2, *A* and *B*), indicating a complete loss of the interaction between ArnA and ArnB under prolonged starvation conditions.

Because FHA domain–containing proteins like ArnA interact generally via phosphorylated threonine residues on their interaction partner, it appears plausible that dephosphorylation of ArnB under nutrient limiting conditions abolishes the interaction. Indeed, several phosphorylated residues have been identified in ArnB previously (24, 25), and thus we investigated the role of the phosphorylated residues of ArnB.

### Crystal structure of ArnA

**Figure 3.**
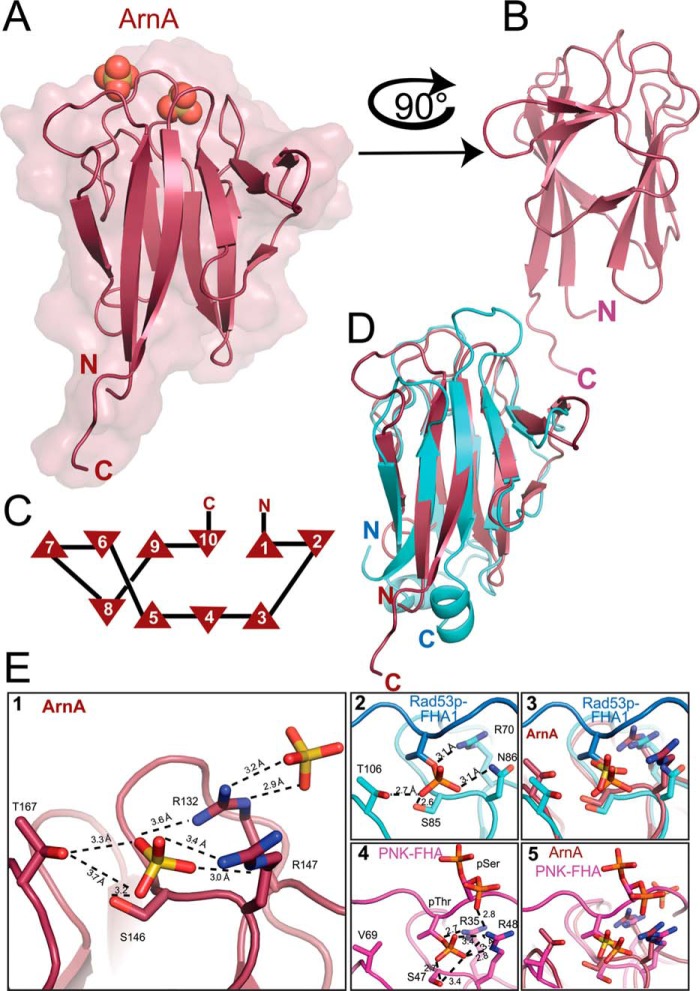
**Crystal structure of the FHA domain from ArnA and cognate phospho-recognition module.** *A*, overview of the FHA domain from ArnA (*red*) in cartoon and surface representation; the two molecules in *spheres* represent the sulfate ions in the structure. *B*, side view of the FHA domain. *C*, secondary structure topology of the FHA domain from ArnA; *triangles* indicate β-strands, and the directions of the *tips* refer to the orientation of the β-strand. *D*, comparison of the FHA domains from the archaeal ArnA (*red*) and the yeast's Rad53p–FHA1 from *S. cerevisiae* (*cyan*) (PDB code 1G6G); the structures superimpose with a r.m.s. deviation of 1.45 Å (61 C_α_ atoms). *E*, cognate phospho-recognition module in ArnA (*panels 1–3*). Primary and (*panels 1*, *4*, and *5*) putative secondary site for phospho-peptide binding (*panels 1*) are shown. *Panel 2*, the structure of ArnA (*red*) exhibits two sulfate ions at its surface; distances are displayed as *dashed lines. Panel 3*, structure of Rad53p–FHA1 from *S. cerevisiae* (*cyan*) (PDB code 1G6G) (9) in complex with a phosphothreonine peptide (*blue*). *Panel 4*, superimposition of ArnA and Rad53p–FHA1; at the position of the phosphate residue in the Rad53p–FHA1 structure, the ArnA structure exhibits the sulfate ion, suggesting a putative phospho-peptide–binding site at this position. The FHA domain of human PNK (*pink*) (PDB code 2W3O) binds a pThr-pSer peptide. pSer exhibits two rotamers in the structure. *Panel 5*, superimposition of ArnA and PNK-FHA.

To better understand the interactions between ArnA and ArnB, we set out to solve their structures. The crystal structure of the FHA domain of ArnA lacking the N-terminal zinc-finger domain (Pro-97–Phe-212) (PDB code 5A8I), which represents the first archaeal FHA crystal structure, was solved by molecular replacement at 1.75 Å resolution (Fig. 3 and Table S4). The structure lacked the N terminus, including the zinc-finger domain (Fig. S1). The FHA domain of ArnA adopted a monomeric 10-stranded β-sandwich fold with four and six β-strands on both sides, respectively (Fig. 3*C*). The N- and C-terminal β-strands of the sandwich, which are the longest β-strands of ArnA, align in an antiparallel fashion, thus bringing the N and C terminus in close proximity. All β-strands are connected by short loop regions on the side of the termini and by longer loops on the opposite side. Here, two sulfate ions are found that originate from the sulfate-containing crystallization condition of ArnA. In the crystal structure of Rad53p–FHA1 from *S. cerevisiae* (Fig. 3*D*), this area complexes a phosphothreonine peptide (Fig. 3*E*) (2). The sulfate ions present in the ArnA structure mimic closely the position of the phosphate group of such a pThr peptide (Fig. 3*E*) (1, 3). This putative phospho-recognition site of ArnA is formed by the same residues as in Rad53p–FHA1: threonine, serine, and arginine (Thr-167, Ser-146, and Arg-132). However, an arginine residue (Arg-147) substitutes an asparagine residue conserved in most FHA domains. Interestingly, in the FHA domain of human PKA, this second arginine residue is found as well and interacts with another pSer. In PKA the affinity for a pSer-Xaa-pThr–containing peptide is nearly 10 times higher than for the pThr-519 peptide (26). In the crystal structure of PKA–FHA, the phosphorylated serine residue of the peptide adopts two rotamers, with one of them hydrogen-bonded to the arginine residue of the primary phospho-peptide–binding site (Fig. 3*E*) (4). Interestingly, in ArnA this arginine of the primary site is involved in binding of a second sulfate ion, suggesting that the electrostatics of the ArnA phospho-peptide–binding site (Fig. S2) allows the binding of a second phosphorylated residue (Fig. 3*E*) (5).

### Crystal structure of vWA2

**Figure 4.**
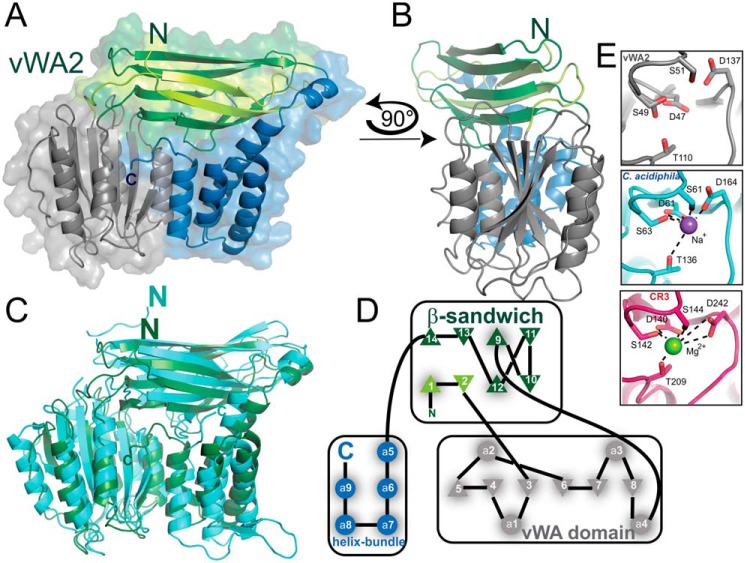
**Crystal structure of vWA2.** *A*, overview of vWA2 in cartoon and surface representation. The vWA domain, the β-sandwich, and the helix bundle are displayed in *gray*, *green*, and *blue*, respectively. The N-terminal β-strands contributing to the β-sandwich are highlighted in *light green. B*, side view of vWA2. *C*, comparison between vWA2 (*green*) and the von Willebrand factor type A (*cyan*) from *C. acidiphila* (PDB code 4FX5) (r.m.s. deviation = 3.750 Å, 264 C_α_ atoms). *D*, secondary structure topology of vWA2. The coloration is based on *A. Triangles* indicate β-strands whose *tips* show the orientation of the β-strand, and *circles* represent α-helices. The β-sandwich exhibits a CnaA-like fold as the N2 domain of the adhesin Sgo0707 (48). *E*, MIDAS motif of vWA2 (*top panel*), the vWA-like gene product from *C. acidiphila* (*middle panel*), and from the α-subunit of the integrin CR3 (PDB code 1IDO) (*bottom panel*) (17).

Because several attempts to crystallize ArnB were unsuccessful, we set out to crystallize Saci_1209 (vWA2), another vWA domain–containing protein of *S. acidocaldarius* and paralog of ArnB (Fig. S1). *S. acidocaldarius* vWA2 and ArnB are not only homologs of each other (sequence identity 31% for 350 residues) but also encoded in close proximity in the genome (upstream and downstream of ArnA) (13). Previously we have shown that (i) contrary to ArnB, no interaction was observed between ArnA and vWA2, (ii) deletion of *vWA2* did not affect the levels of FlaB and FlaX, and (iii) deletion of *vWA2* did not affect motility on plate, suggesting that vWA2 is not directly involved in motility regulation (13). Indeed, overexpression of *vWA2* failed to complement the hypermotile phenotype of the *arnB* deletion mutant (Fig. S3), thus excluding the possibility that vWA2 is directly involved in the regulation of motility. However, given their homology, the structures of vWA2 and ArnB are expected to resemble each other and make vWA2 to a valid model for ArnB apart from a missing 20-amino acid C-terminal extension that is exclusively part of ArnB (Fig. S1). The complete structure of vWA2 (Ala-2–Gly-366) (PDB code 5A8J) was determined from an orthorhombic crystal form at a resolution of 1.45 Å *via* MAD phasing. The vWA2 structure represents the first archaeal example of a vWA-containing gene product (Fig. 4, *A* and *B*; Fig. S1; and Table S4) and harbors in addition to the vWA-domain a β-sandwich and a four-helix bundle. The vWA domain is built of a central, twisted six-stranded β-sheet flanked by four helices with largely alternating α-helices and β-strands. Interestingly, the complex topology of vWA2/ArnB implies that the vWA domain is inserted within the eight-stranded β-sandwich. Here, six of the eight β-strands (strands CDD′EFG) are formed by a stretch next to the C terminus of the vWA domain, whereas the other two β-strands (AB) are derived from the very N terminus of vWA2 (Fig. 4, *A* and *D*). With its two antiparallel β-sheets, ABED and D′CFG, this β-sandwich is topologically related to a distorted C-type immunoglobulin domain. Here, at the C terminus, the vWA2 structure is completed by a four-helix bundle that spatially connects the vWA domain and the β-sandwich structure which are edge-to-edge oriented toward each other. The same tripartite structure of vWA2 is found for the structure of a von Willebrand factor type A–like gene product from *Catenulispora acidiphila*, a bacterium belonging to the actinobacterial class (PDB code 4FX5; gene name: *caci_2163*; r.m.s. deviation 3.75 Å for 264 C_α_-atoms). Although the overall sequence identity is marginal, *i.e.* only 20% for amino acids Ile-57–Asn-222, which represent the vWA domain, the structural similarity between vWA2 and the vWA-like gene product from *C. acidiphila* is high including the 8-stranded β-sandwich domain and the C-terminal four-helical bundle (Fig. 4*C*). Furthermore, ArnB, vWA2, and the vWA-like gene product from *C. acidiphila* harbor an intact MIDAS (Fig. 4*E*) in the vWA domain. Although no Mg^2+^ or Ca^2+^ ion is found in the vWA2 structure, the structure of the *C. acidiphila* ortholog harbors here a bound sodium ion (Fig. 4*E*). Overall, we solved the first structures of an archaeal FHA domain–containing protein, as well as of an archaeal vWA2 domain–containing gene product.

### Identification of possible sites of interaction between ArnA and ArnB

**Figure 5.**
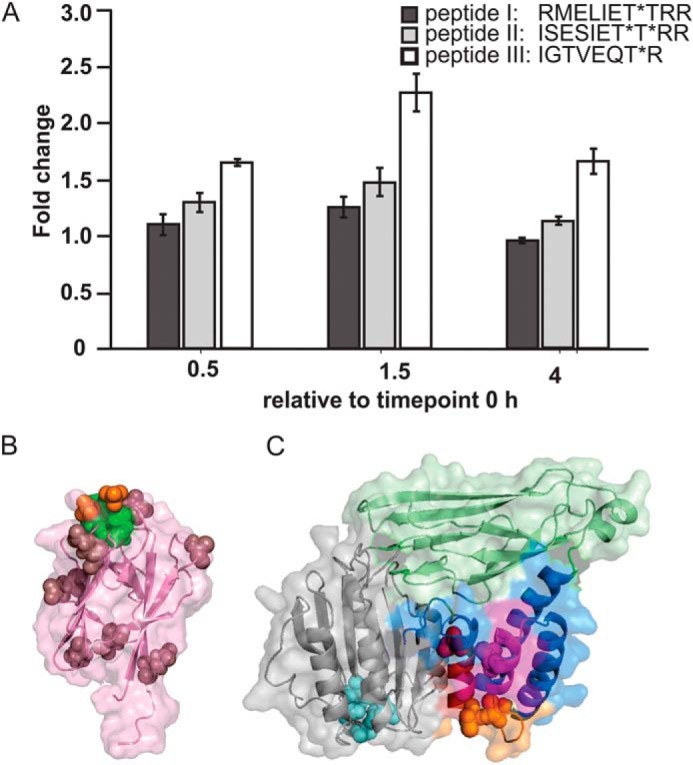
**The C terminus of ArnB is phosphorylated.** *A*, changed abundances of phosphorylated peptides detected from ArnB during nutrient limitation. The data were compared with time = 0 h (before nutrient limitation). *B*, structure of ArnA highlighting the positions of surface-exposed glutamates (*purple*) and the site where a phosphothreonine peptide (*red* and *green spheres*) might bind. *C*, structural model of ArnB and localization of *in vitro* phosphorylated amino acids. The homology model was generated by SWISS-MODEL with vWA2 as template (42, 43) and shown in the same orientation as vWA2 in Fig. 4. The vWA domain is represented in *gray*, and the amino acids of its characteristic metal ion adhesion site (MIDAS) are in *cyan*. Phosphorylated residues identified by ITRAQ analysis and in a previous study (24) are depicted: *red*, pThr of peptide 1 (RMELIET*T*RR); *magenta*, pThr of peptide 2 (ISESIET*T*RR); and *yellow*, pThr of peptide 3 (IDT*VEQT*R).

To assess whether the phosphorylation pattern of ArnB is altered in the course of starvation, *S. acidocaldarius* was cultured to early exponential phase in nutrient-rich conditions (0 h) and transferred to medium lacking nutrients and analyzed at 0.5, 1.5, and 4 h after nutrient depletion. Then iTRAQ analysis was performed to identify and quantify phosphorylated peptides from the ArnB protein. As a result, three unique phosphorylated peptides derived from ArnB (RMELIET*T*RR, ISESIET*T*RR, and IGTVEQT*R) were detected and quantified (Fig. 5*A*). These three consecutive peptides (I–III: Arg-337–Arg-346, Ile-347–Arg-356, Ile-357–Arg-364) were also identified as three of the four highest phosphorylated peptides by *in vitro* phosphorylation experiments, when ArnB was phosphorylated by ArnC or ArnD, respectively (24). Remarkably, the phosphorylated peptides derived from the very C terminus of ArnB (EIT*S*EVT*KK and EITS*EVT*KK, Glu-368–Lys-377), which were detected during *in vitro* experiments with highest intensities, could not be detected *in vivo* at significant levels (24). As shown in Fig. 5*A*, the abundance of two of the three preceding phosphorylated peptides, modulated only slightly during nutrient starvation. In contrast, the abundance of peptide III shows an increase up to 2.3-fold after 1.5 h, before going slightly down at 4 h. Remarkably, no peptides derived from ArnB could be detected by our iTRAQ analysis, in which a decrease in the phosphorylation levels correlated with the loss of interaction between ArnA and ArnB during starvation.

As noted, upon nutrient depletion, the ArnA–ArnB interaction is gradually abolished when the expression levels of the archaellum increases (Fig. 2). Our structural analysis identified several potential sites of interaction in ArnA and ArnB. The MIDAS site of ArnB, which resembles the MIDAS sites of α6β1 integrin (19) and midasin (20), might interact with a glutamate residue from an interacting partner. Fig. 5*B* shows the positions of surface-located glutamate residues of ArnA. Furthermore, the phospho-recognition site of ArnA, which is also indicated in Fig. 5*B*, might interact with phosphorylated residues of ArnB. Several phosphorylated residues have been identified in ArnB previously (24). The threonine residues found in our iTRAQ analysis were also identified in the previous study, and also other threonine residues, which are located within the three peptides identified by iTRAQ. Fig. 5*C* shows the positions of all phosphorylated threonine residues that were identified in both studies, as mapped on a vWA2-derived homology model of ArnB. Remarkably, all identified phosphorylated residues are located in the C-terminal four-helix bundle of ArnB. We substituted the phosphorylated threonine residues pairwise by alanine in an order following that of the three peptides that were identified by iTRAQ (T343A/T344A, T353A/T354A, and T359A/T363A) to assess whether the phosphorylation of these residues is (i) important for the function of ArnB in regulation of motility and (ii) involved in the interaction of ArnA with ArnB.

**Figure 6.**
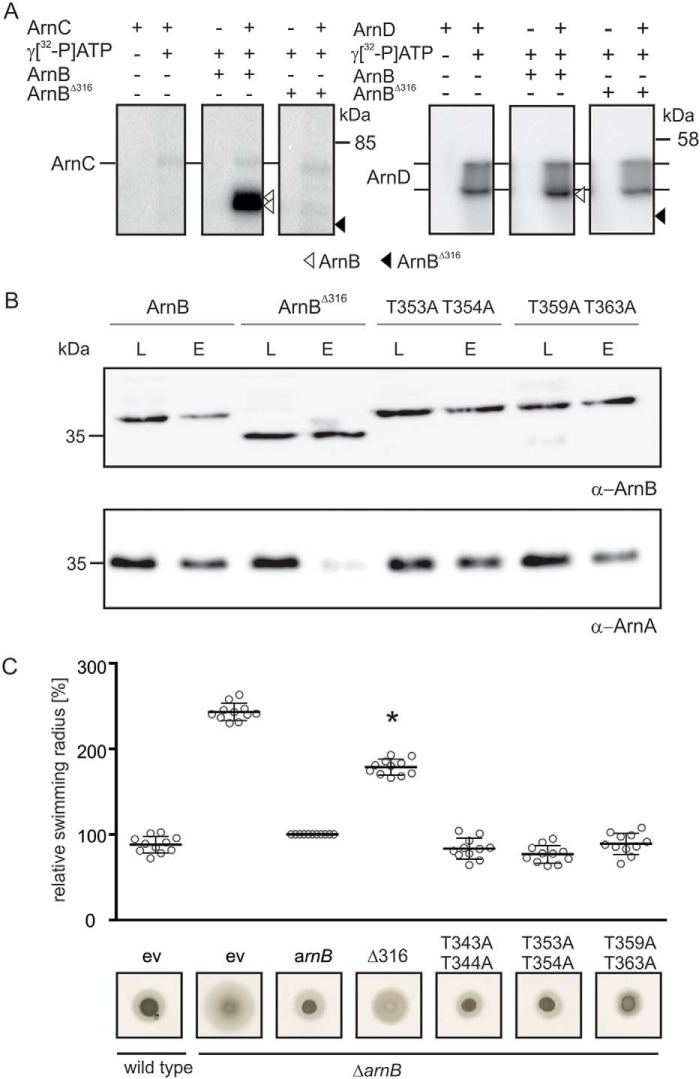
**The C terminus of ArnB is crucial for ArnA–ArnB interaction.** *A*, *in vitro* phosphorylation of ArnB and ArnB^Δ316^ with the Ser/Thr kinase ArnC. ArnC autophosphorylates upon incubation with [γ-^32^P]ATP (*left panels*). Phosphotransfer from ArnC to WT ArnB (*middle panels*) but not ArnB^Δ316^ (*right panels*) was observed. The assays were repeated three times, and a representative phosphorimage is depicted. *B*, *in vivo* interaction of ArnA with Strep/His-ArnB, Strep/His-ArnB^Δ316^, and Strep-HisArnB phosphorylation ablative mutants. Western blotting analyses of the load (*L*) and elution (*E*) fractions obtained during pulldown analysis with Streptactin-coated magnetic beads were performed with ArnA and ArnB specific primary antibodies. *C*, swimming radius and motility assay of Δ*arnB* and Δ*arnB* complementation with the different ArnB mutants. Plasmid encoded *arnB*^Δ316^, *arnB T343A/T344A*, *arnB T353A/T354A*, and *arnB T359A/T363A* were used to complement the Δ*arnB* strain, and their swimming radii were subsequently compared with Δ*arnB* cells complemented with WT *arnB*. All strains were analyzed for their motility phenotype on semi-solid plates. After growth for 4 days at 75 °C, the swimming radii of the strains were calculated. *Bars* represent the mean relative swimming radius of three independent biological replicates with each six technical replicates normalized to the Δ*arnB* strain complemented with WT *arnB* in *trans*, which was set to 100%. Statistical significance was analyzed using a Student's *t* test (unequal variance, two-tailed) compared with the Δ*arnB* strain complemented with WT *arnB. p* values of <0.05 are indicated by *asterisks*.

To further study whether the C-terminal four-helix bundle of ArnB is important for the interaction with ArnA, ArnB was truncated after Asn-316, resulting in ArnB^Δ316^, which no longer contained most of the phosphorylated serine and threonine residues (24). *In vitro* phosphorylation assays were performed with [γ-^32^P]ATP and the S/T kinases ArnC and ArnD (Fig. 6*A*). Upon incubation with [γ-^32^P]ATP, autophosphorylation of the respective kinase, and upon addition of ArnB, phosphotransfer to ArnB was detected. In contrast, no phosphotransfer was found to occur to ArnB^Δ316^ (Fig. 6*A*), supporting the previous notion that phosphorylated residues are predominantly located at the C terminus of ArnB (24).

To analyze whether ArnB T343A/T344A, ArnB T353A/T354A, ArnB T359A/T363A, and ArnB^Δ316^ were still able to interact with ArnA, pulldown experiments with plasmid-encoded Strep-tagged ArnB WT and mutant variants were performed (Fig. 6*B*). All ArnB variants were expressed in *S. acidocaldarius* ΔArnB using a maltose-inducible promoter (27) and were grown in Brock medium supplemented with maltose to induce protein expression.

Because it was previously observed that the *in vivo* stability of another archaellum regulator AbfR1 depends on its phosphorylation state, we assessed levels of plasmid-expressed ArnB WT and ArnB mutants and found that all proteins were expressed and stable (Fig. S4). However, unexpectedly ArnB T343A/T344A did not bind to the StrepMag beads (Fig. S5), which hindered the analysis of the interaction with ArnA in this mutant. Interaction of ArnA with the other alanine mutants was observed, because ArnA was detected in elution fractions obtained from all pulldown experiments of cells grown to early exponential phase. (Fig. 6*B*). Strikingly, co-elution of ArnA with ArnB^Δ316^ was strongly reduced compared with co-elution of ArnA with WT ArnB, demonstrating that the C terminus of ArnB is important for the interaction with ArnA. To test the influence of the mutations and the C-terminal deletion of ArnB on motility of *S. acidocaldarius*, motility assays were performed (Fig. 6*C*). As observed previously, deletion of *arnB* resulted in hypermotility, a phenotype that could be complemented by the expression of plasmid-encoded ArnB (13) (Fig. 6*C*). Although plasmid-based expression of all alanine mutant variants, including the T343A/T344A mutant, which we were unable to test in the interaction assay above, showed swimming behavior similar to that of the in *trans* complemented *arnB* deletion mutant, expression of plasmid-encoded ArnB^Δ316^ failed to fully complement the hypermotility phenotype (Fig. 6*C*). Thus, our data demonstrate that the C-terminal four-helix bundle of ArnB is essential for the interaction with ArnA. This C-terminal domain can be phosphorylated by ArnC *in vitro* and is found phosphorylated *in vivo*, suggesting that phosphorylation of this domain might influence its interaction with ArnA. However, mutation of the single identified phosphorylated residues did not affect motility, suggesting that multiple residues might play a role in this process

## Discussion

So far little is known about signal transduction in archaea. We have previously shown that deletion of the genes encoding the protein kinases *saci_0965* and *arnC* (24), as well as ArnS (28), resulted in reduced motility, whereas deletion of the protein kinase encoding gene *arnD* (24) and the phosphatase PP2A (25) results in strongly increased motility in nutrient-limiting conditions in *S. acidocaldarius*. These proteins are part of a regulatory network, called the archaellum regulatory network (arn). Indeed, several of the components of this network in *S. acidocaldarius,* like ArnA, ArnB (13, 25), and AbfR1, a transcriptional regulator of the Lrs14-type, are subjected to phosphorylation (25). In *Sulfolobus solfataricus*, the main activator of expression of the archaellum, ArnR was also found to be phosphorylated (29). Thus, motility in *Sulfolobales* is very likely regulated via phosphorylation. To our knowledge, the archaellum regulatory network is hence the first archaeal signal transduction cascade involving S/T/Y phosphorylation that is described in some detail.

Here we set out to characterize the FHA domain–containing protein ArnA and the vWA domain–containing protein ArnB. As previously shown, deletion of *arnA* and/or *arnB* resulted in even higher expression levels of FlaB and FlaX under starvation conditions, more archaella on the surface, and hypermotility on plate, but deletion of *arnA* and *arnB* had no strong effects during growth on rich medium (13). This demonstrated that ArnA and ArnB are involved in tuning the expression levels under nutrient limiting conditions, and it was suggested that this might occur via an increase in the ArnA and ArnB levels during starvation. Remarkably, in this study no significant changes could be observed for ArnA and ArnB levels when cells were transferred to starvation conditions. This demonstrated that the regulation of the expression levels of the archaellum components is independent of changes of the ArnA and ArnB levels. Several observations are consistent with the hypothesis that the ArnA–ArnB interaction is crucial for regulating the expression of the archaellum components and that this interaction becomes changed in a phosphorylation-dependent manner (13, 24): (i) ArnA and ArnB strongly interact with each other (13), (ii) deletion mutants of ArnA and ArnB exhibit similar phenotypes (13), (iii) ArnA and ArnB have been detected in phosphorylated states in *S. acidocaldarius* (25), (iv) recombinant ArnC and ArnD phosphorylate serine and threonine residues in the C terminus of ArnB *in vitro* (24), and (v) ArnA contains a phospho-recognition module (13).

Indeed, we found that the strong interaction between ArnA and ArnB, which was observed during growth on rich medium, was sequentially diminished after nutrient limitation. Furthermore, a mutant lacking the C-terminal domain, in which the phosphorylated residues were identified and in which this interaction was strongly reduced (ArnBΔ^316^), could only partly complement the Δ*arnB* strain, further suggesting that the ArnA–ArnB interaction plays an important role in the regulation of archaellum expression.

To study this further, the structures of ArnA and the ArnB homolog vWA2 were determined. Our ArnA structure only shows the C-terminal FHA domain because the N-terminal domain containing the zinc-finger motif was apparently proteolytically cleaved off during crystallization. This N-terminal domain contains a RanBP2-type zinc-finger motif, in which a single Zn^2+^ ion is coordinated with four cysteine residues located in two orthogonal β-hairpin strands. Members of this zinc-finger domain family have been shown to interact with other proteins and with RNA (30, 31). The FHA domain of ArnA shows a similar fold and conservation of amino acids in the phosphate-recognition module as the well-characterized FHA-1 domain of Rad53p from *S. cerevisiae* (32) or bacterial homologs like GarA and RV1747 from *Mycobacterium tuberculosis* (33, 34). The binding site that is specific for pThr recognition and harbors a threonine or aspartate, a serine, and an arginine residue, which are located in the loops between β3-β4 and β5-β6 (9) and are likewise present in the structure of ArnA. This indicates that ArnA is indeed capable of recognizing pThr-containing signature motifs, suggesting that the FHA domains of Crenarchaeota function like their bacterial and eukaryotic counterparts. Further evidence for this assumption provides the modeled structure of St0829, an FHA domain-containing protein of *S. tokodaii,* where residues crucial for pThr recognition are conserved as in ArnA. Moreover, mutation of any of these residues affected the function of St0829 *in vitro* (11) analogous to the phospho-recognition site of Rad53p–FHA1 (32), suggesting that ArnA binds phosphorylated threonines. Furthermore, the structure of ArnA contained two sulfate ions, whose positions suggest that ArnA is able to bind double-phosphorylated peptides (Fig. 5*A*). Most FHA domain–containing proteins studied so far usually recognize only a single pThr residue. Interestingly, several FHA domains like that one in the Dun1 kinase of *S. cerevisiae* bind to a duplicated threonine–phosphate recognition motif (pT*XX*pT*XX*S) with higher affinity than the monophosphorylated motif (35), suggesting that a recognition motif in ArnB might also consist of multiple residues.

The crystal structure of the ArnB homolog vWA2 consists of three domains: a vWA domain, a β-sandwich domain, and a four-helix bundle domain. The vWA domain revealed an intact MIDAS site, which might be involved in interaction with a glutamate residue coming from the interacting partner. The C-terminal domain four-helix bundle contained almost all the previously identified phosphorylated residues (24). Indeed, several of these residues are lying close enough to function as the double phosphorylated interaction partner for ArnA. Remarkably, these motifs are not easily accessible in the ArnB structure modeled on the vWA2 structure (Fig. 5*C*), suggesting that conformational changes must occur before these motifs can be phosphorylated and that phosphorylation of these residues will also lead to conformational changes within ArnB. Indeed, the C-terminal helix bundle of ArnB includes a helix-turn-helix motif that forms only weak, secondary interactions with the adjacent β-sandwich like a salt bridge between Arg-24 and Glu-299 and weak hydrophobic interactions in the small interface. This supports a signaling switch model, in which phosphorylation of the C terminus of ArnB is coupled to conformational changes.

Overall, our structures indicate that ArnA contains a functional phospho-recognition motif that might recognize several phosphorylated motifs in the highly phosphorylated C-terminal four-helix bundle domain of ArnB. Further possible interactions might occur via the MIDAS site in ArnB and the N-terminal zinc-finger domain of ArnA. Together with our other data, these results suggest that under rich conditions ArnA and ArnB might interact via the phospho-recognition module of ArnA and the phosphorylated four-helix bundle domain of ArnB and that under nutrient depleting conditions ArnB becomes dephosphorylated at these residues, and in consequence the interaction between ArnA and ArnB is lost. To assess whether the phosphorylation pattern of ArnB is altered after nutrient depletion, we performed iTRAQ analysis and quantified several phosphorylated peptides of ArnB. However, no peptides could be detected, where such a decrease in the phosphorylation levels was found, which can be correlated with the observed loss of ArnA–ArnB interaction during nutrient depletion. Furthermore, mutation of pairs of the identified phosphorylated residues did not affect motility or the interaction between ArnA and ArnB.

Accordingly, either multiple phosphorylated residues might play a role in this process or under starvation conditions a currently unidentified protein may become phosphorylated and recruit ArnA by displacing ArnB. In this context it is also plausible that ArnB may interact with other cellular components given its prominent electrostatic properties (Fig. S2). For example, ArnB exhibits patches of negative potentials at the vWA domain and a positive potential patch on the side of the β sandwich, as well as a negative deepening on top of it. In the case of an interaction, it will be interesting to know whether the different phosphorylation patterns of ArnB allow interaction with different partners necessary for motility regulation. The identification of these interaction partners for example by immune-precipitation and furthermore the roles of the MIDAS and zinc-finger domains in the interaction of ArnA and ArnB will require further studies of this motility repression module.

## Experimental procedures

### S. acidocaldarius strains, plasmids, and growth conditions

The uracil auxotroph *S. acidocaldarius* MW001 was used as background strain for the generation of markerless in-frame deletion mutants. All strains used in this study are described in Table S1. Strains were grown essentially as described, using basal Brock medium (pH 3.5) supplemented with 0.1% NZ-amine, 0.2% sucrose, and 10 μg/ml uracil (27). Methylated plasmids were transformed with *S. acidocaldarius*, as described (27). Transformed cells were recovered at 75 °C for 30 min in basal Brock medium containing 0.1% NZ-amine and 0.2% sucrose and plated on Gelrite plates (0.6% Gelrite) supplemented with 0.1% NZ-amine and 0.2% sucrose. The plates were incubated at 75 °C for 5 days. Successful transformation was controlled by PCR. Uracil was not added to the medium of strains that were complemented in *trans* because utilized plasmids encode the *pyrEF* cassette. Plasmids used for in *trans* complementation are described in Table S2. Primers used for their construction are described in Table S3.

### Escherichia coli strains, plasmids, and growth conditions

All *E. coli* strains used in this study are described in Table S1. Plasmids used in this study and their creation are described in Table S2. Primers used for their construction are describe in Table S3. All strains were grown in LB medium supplemented with 0.2% glucose and 50 μg/ml ampicillin. In case of BL21(DE3)RIL and Rosetta(DE3)pLysS strains, 30 μg/ml chloramphenicol was added to the medium.

### Expression and purification of recombinant proteins from E. coli

Expression of ArnB and ArnB^Δ316^ was performed in *E. coli* BL21 RIL. vWA2 (saci1209) and ArnA (saci1210) was expressed and purified essentially as described (13). Heterologous gene expression in *E. coli* and processing of cells were performed essentially as described (13), including heat precipitation of *E. coli* proteins from the cell lysate at 70 °C for 10 min followed by an additional centrifugation step at 19,000 × *g* and 4 °C for 30 min (Beckmann Coulter Optima MAX-XP, rotor MLA55). The supernatant was applied for His-tag affinity purification on His-select nickel-affinity gel (Sigma). Proteins were eluted in 20 mm HEPES/NaOH, pH 7, 100 mm KCl, 100 mm imidazole. Strep-tag affinity purification of ArnA was performed using a Strep-tactin superflow column (IBA) in 100 mm Tris, pH 8.0, 150 mm NaCl, 2.5 mm desthiobiotin. Finally, affinity-purified vWA2 and ArnA were subjected to size-exclusion chromatography (Superdex 200 16/60, GE Healthcare) in 20 mm HEPES, pH 7.0, 100 mm KCl.

### Crystallization and data collection

Purified recombinant ArnA (protein concentration, 3.5 mg/ml) in 20 mm Tris, 100 mm NaCl, pH 8.0, was crystallized using the hanging-drop vapor-diffusion method. 1 μl of protein was added to 1 μl of reservoir solution (0.1 m HEPES, pH 7.5, 1.5 m Li_2_SO_4_). After 2 months at 18 °C a crystal appeared, which was frozen in reservoir buffer supplemented with 30% (v/v) glycerol for data collection. The data set was recorded at Beamline 14.1 (Berliner Elektronenspeicherring–Gesellschaft für Synchrotronstrahlung, BESSY-II, Berlin, Germany). Initial phasing was performed with molecular replacement using the CCP4 package (36, 37). The starting model was generated by Modeler (38) using EmbR, a FHA domain–containing protein (PDB code 2FF4; chain A), as template structure. Structural refinement using the 1.75 Å data set was performed with PHENIX (39) and COOT (40). Structure diagrams were created using PyMOL 0.99 (41). The crystal for the native data set of vWA2 was grown at 18 °C in drops that contained 0.3 μl of the protein (2.7 mg/ml) in 20 mm HEPES, pH 7.0, 100 mm KCl, and 0.3 μl of the reservoir solution (0.1 m sodium citrate, pH 5.5, 2.5 m (NH_4_)_2_SO_4_). The crystal for the MAD data sets appeared in a drop containing 0.3 μl of the protein in HEPES buffer at a concentration of 4.0 mg/ml and 0.3 μl of reservoir solution (0.2 m K_3_PO_4_, 2.2 m (NH_4_)_2_SO_4_). The crystals were soaked for 1 h with 50 mm Na_2_WO_4_. For data collection, native and heavy metal–supplemented crystals were frozen in reservoir solution containing 30% (v/v) glycerol. All data sets were recorded at the European Synchrotron Radiation Facility in Grenoble, France, with the native data set at Beamline ID29 and the 2.0-Å MAD data sets at Beamline ID14-4. Initial MAD phasing attributable to a Keggin cluster formed by tungstate-phosphate was performed with the CCP4 package, and the structural refinement of the 1.45-Å native data set was performed with PHENIX. The homology model of ArnB based on the vWA2 structure was generated with SWISS-MODEL (42, 43).

### Motility assay

Motility assays were performed as described (23, 24). The plates were scanned, and the swimming radius of at least 18 individual colonies was measured according to Ref. 24.

### In vitro phosphorylation assays

*In vitro* phosphorylation assays using [γ-^32^P]ATP (Hartmann Analytic) were performed as described (13). For phosphorylation of purified ArnB and ArnB^Δ316^, the kinases ArnC and ArnD were used. 2 μm of protein was mixed with 2 μm of ArnC or 0.2 μm of ArnD in a 5× reaction buffer. For assays with ArnD the reaction buffer contained 125 mm MES, pH 6.5, and 750 mm KCl. For assays with ArnC the buffer contained 250 mm HEPES, pH 7.8, and 750 mm KCl. Finally, a mix consisting of 0.8 mm nonradioactive ATP and 0.3 mm [γ-^32^P]ATP was added to the reaction. After incubation at 55 °C for 10 min, proteins were separated on 11% SDS gels and exposed on a phosphostorage screen (Molecular Dynamics) overnight. Screens were scanned using a phosphorimaging device (Storm 840, Molecular Dynamics).

### Western blotting analysis

Comparison of protein amounts in cells under nutrient-rich and nutrient-depleted conditions was performed as described (24). Samples of whole cells were pelleted, resuspended to an A_600_ of 10 in 1× PBS, and supplemented with SDS-loading buffer. After incubation at 100 °C for 10 min 10 μl/sample were subjected to SDS-PAGE analysis according to the method of Laemmli using 11% gels (44).

Subsequently, proteins were transferred onto polyvinylidene difluoride membranes (Roche Diagnostics) using the semi-dry method (45). FlaB was detected as described (23). Primary polyclonal antibodies specific for ArnA and ArnB were raised in rabbits (Eurogentec) and used as primary antibodies in a dilution of 1:5000 in PBST. HRP-coupled anti-rabbit IgG (Invitrogen) diluted 1:10.000 in 1× PBST was used as a secondary antibody, and chemiluminescent signals were detected as described (24). Obtained signals were quantified using ImageJ.

### Pulldown assay with magnetic Strep beads

Pulldown assays of Strep–ArnB, Strep–His–ArnB, Strep–His–ArnB mutants, and ArnA were performed using MagStrep type 3 XT beads (IBA) according to the manufacturer's protocol. For each assay 200-ml samples of cultures either grown to exponential phase (*A*_600_ = ∼0.4) or cultures that were grown in nutrient-depleted medium for either 0.5, 1.5, or 4 h were collected as described (24). For the interaction analysis of plasmid-encoded Strep–His–ArnB and mutant variants with ArnA, 0.2% maltose was added to the growth medium instead of 0.2% dextrin to induce protein expression. After cell harvesting, 1 ml of wash buffer was added per 100 mg of pellet weight. Subsequently the cells were disrupted by sonication (30% intensity, 10 min, 15-s intervals, Bandelin Sonoplus). Cell debris was removed by centrifugation. 20 μl of magnetic bead solution (corresponding to 1 μl of magnetic beads) per 100 mg of pellet weight was prepared according to manufacturer's protocol. A sample of the load and elution fraction was collected, and 15 μl of each fraction was analyzed by SDS-PAGE and subsequent Western blotting with ArnA- and ArnB-specific antibodies as described above.

### Protein extraction and iTRAQ labeling

Frozen cells of *S. acidocaldarius* MW001 collected at 0 h (before nutrient limitation) and at 0.5, 1.5, and 4 h after starvation were washed with ice-cold water before being resuspended in 1 ml of protein extraction buffer consisting of 0.05% SDS in 0.5 m triethylammonium bicarbonate, pH 8.5. Protein extraction was carried out using an ultrasonicator (Sonifier 450, Branson) for 8 times at 70% duty cycle (alternatively 45 s of sonication and 45 s on ice). Extracted proteins were collected by centrifugation at 21,000 × *g* at 4 °C for 30 min (Heraeus Multifuge, Thermo), and protein concentrations were determined using a Bradford assay (Sigma). A total of 100 μg of proteins from each sample (two biological replicates for each sampling point) was used for an 8-plex iTRAQ analysis with 8 iTRAQ tags used, and the analysis was performed based on the manufacturer's instruction. Briefly, these proteins were reduced by 2 μl of 50 mm tris-(2-carboxyethyl) phosphine at 60 °C for 1 h, and alkylated by 1 μl of 200 mm methyl methanethiosulfonate for 10 min at room temperature before being digested by trypsin MS grade (Promega) with the ratio of trypsin:proteins 1:20. Digested proteins from four different sampling points were then labeled with iTRAQ reagents as follows: 0 h, 113 and 114 (control); 0.5 h, 115 and 116; 1.5 h, 117 and 118; and 4 h, 119 and 121, and incubated at room temperature for 2 h. All labeled peptides were then combined before being dried in a vacuum concentrator (Eppendorf Concentrator 5301).

### Labeled peptides fractionation

Hydrophilic interaction chromatography (HILIC) was used for cleaning and fractionation of dried iTRAQ-labeled peptides, in which peptides were resuspended in 100 μl of HILIC buffer A containing 10 mm ammonium formate in 80% acetonitrile (ACN), pH 3.0, before being loading onto a 4.6 × 200-mm PolyHYDROXYETHYL-A column (5 μm, 200 Å, Hichrom Limited) coupled with an ultra-high performance liquid chromatography 3000 system (Dionex). An UV detector was used to monitor peptides' abundance at a wavelength of 280 nm. Peptides were fractionated at a flow rate of 0.5 ml/min using a gradient with HILIC buffer B containing 10 mm ammonium formate in 5% ACN, pH 5.0; 10 min of 2% buffer B before ramping up to 20% of buffer B for 5 min, and to 60% of buffer B for 50 min, and hen ramped up to 100% of buffer B for 10 min and kept for 10 min, and finally 0% of buffer B for 5 min. Eluted peptides were collected every 2 min and then dried in a vacuum concentrator before being cleaned using C_18_ spinning tips (Nest Group) before submitting to a mass spectrometer.

### Nano LC-MS/MS analysis

Selected cleaned peptides (from different fractions) were redissolved in 20 μl of buffer A consisting of 0.1% formic acid in 3% ACN then combined into six different fractions before 3 μl of sample was withdrawn and submitted onto a Q Exactive^TM^ hybrid quadrupole-Orbitrap mass spectrometer (Thermo, Germany) coupled with a nano ultra-high performance liquid chromatography 3000 system (Dinonex) operated at a flow rate of 0.3 μl/min. Peptides were separated using a C18 column with a 105-min gradient of buffer B (0.1% formic acid in 97% ACN) as follow: 3% for 5 min, then ramped up to 10% for 5 min, 50% for 75 min, 90% for 1 min, then kept at 90% for 4 min before being ramped back to 3% buffer B for 1 min, and then maintained at 3% for 14 min. The MS was operated in positive mode with resolutions of full MS and ddMS set at 60,000 and 15,000, respectively. AGC targets were set at 3.106 and 5.104 for full MS and ddMS, respectively; maximum IT times were set at 100 and 20 ms for full MS and ddMS, respectively; a full mass scan ranging from 375 to 1500 *m*/*z* was applied for MS, whereas the mass scan of 100–1500 *m*/*z* was applied for ddMS; default charge state of ion was set at 2.

### Identification and quantitation of peptides/proteins

All raw data files from MS analysis were submitted to MaxQuant version 1.5.3.8 for protein identification against the *S. acidocaldarius* MW001 database (consisting of 2,361 entries). Modifications of iTRAQ reagents (on N-terminal and lysine residue) and methylmethanethiosulfate were set as fixed modifications, whereas methionine oxidation and phosphorylation (Ser, Thr, and Tyr) were set as variable modification; trypsin digestion used with a maximum missed cleavages of 2; a minimum peptide length of 6, and a maximum peptide mass of 4600 Da were set; tolerances of 20 and 4.5 ppm were applied for MS and MS/MS, respectively. A false discovery rate of 0.01 was used for identification of both peptides and proteins; a minimum score of 40 was used for modified peptides. All detected peptides containing intensities of iTRAQ reagents, from MaxQuant, were then submitted to an in-house proteomic pipeline for quantitation of protein and determination of regulated proteins (46, 47). First, data imported from MaxQuant was filtered to remove both reversed and potential commination peptides, and then proteins identified/quantified by a single peptide were removed before intensities of iTRAQ reporters of peptides being transformed into ln form for further analyses. The quantitation was also done using mean and isobaric corrections. *t* tests (α = 0.01) were then performed at the peptide level (peptides corresponding to an identified protein) to determine regulated proteins for each phenotype comparison. Phosphorylated peptides were manually examined and calculated for their relative ratios with time, *t* = 0 h used as a control.

## Author contributions

L. H., K. A., L. F. B., J. R., S. K., and T. K. P. data curation; L. H., K. A., L. F. B., X. Y., J. R., S. K., T. K. P., and C. v. d. D. formal analysis; L. H., K. A., L. F. B., T. K. P., and S.-V. A. writing-original draft; K. A., L. F. B., X. Y., J. R., T. K. P., and C. v. d. D. investigation; X. Y. funding acquisition; T. K. P., C. v. d. D., and P. C. W. methodology; C. v. d. D., P. C. W., L.-O. E., and S.-V. A. supervision; C. v. d. D., P. C. W., L.-O. E., and S.-V. A. writing-review and editing; L.-O. E. and S.-V. A. resources; L.-O. E. and S.-V. A. project administration; S.-V. A. conceptualization; S.-V. A. visualization.

## Supplementary Material

Supporting Information

## Footnotes

4 The abbreviations used are:

FHA: forkhead-associated
vWA: von Willebrand type A
PDB: Protein Data Bank
MIDAS: metal ion–dependent adhesion site
Strep: streptavidin
PKA: protein kinase A
r.m.s.: root mean square
HILIC: hydrophilic interaction chromatography
ACN: acetonitrile
ddMS2: data dependent MS/MS.
