# Supplementary material for: Structure and interactions of the archaeal motility repression module ArnA–ArnB that modulates archaellum gene expression in Sulfolobus acidocaldarius
Source: J Biol Chem. 2019 Mar 22;294(18):7460–71. doi: 10.1074/jbc.RA119.007709 (PMC6509490; doi:10.1074/jbc.RA119.007709)
Supplement: Supporting Information [file supp_294_18_7460__index.html]

Structure and interactions of the archaeal motility repression module ArnA-ArnB that modulates archaellum gene expression in Sulfolobus acidocaldarius — Characterization of ArnA and ArnB — Structure and interactions of the archaeal motility repression module ArnA–ArnB that modulates archaellum gene expression in Sulfolobus acidocaldarius — Characterization of ArnA and ArnB — Supporting Information 

# Structure and interactions of the archaeal motility repression module ArnA–ArnB that modulates archaellum gene expression in *Sulfolobus acidocaldarius*

## Supporting Information

- Supporting Information (to be published online) - Supplementary material
