# Supplementary material for: Structure and interactions of the archaeal motility repression module ArnA–ArnB that modulates archaellum gene expression in Sulfolobus acidocaldarius
Source: J Biol Chem. 2019 Mar 22;294(18):7460–71. doi: 10.1074/jbc.RA119.007709 (PMC6509490; doi:10.1074/jbc.RA119.007709)
Supplement: Supporting Information [file supp_RA119.007709_143339_1_supp_304623_phs0fn.docx]

**Supplementary information to:**

**“Structure and interactions of the archaeal motility repression module ArnA-ArnB that modulates archaellum gene expression in *Sulfolobus acidocaldarius”***

Lena Hoffmann^1#^, Katrin Anders^2#^, Lisa F. Bischof^1,3,#^, Xing Ye^1^, Julia Reimann^1^, Sunia Khadouma^1^, Trong K. Pham^5^, Chris van der Does^1^, Phillip C. Wright^7^, Lars-Oliver Essen^2,4*^ & Sonja-Verena Albers^1*^

Running title: Characterization of ArnA and ArnB

^1^University of Freiburg, Faculty of Biology, Molecular Biology of Archaea, Schaenzlestrasse 1, 79104 Freiburg, Germany; ^2^Philipps University, Department of Chemistry, Hans-Meerwein-Strasse 4, 35032 Marburg, Germany, ^3^Spemann Graduate School of Biology and Medicine (SGBM), University of Freiburg, ^4^LOEWE Center for Synthetic Microbiology, Marburg, ^5^ChELSI Institute, Department of Chemical and Biological Engineering, the University of Sheffield, Mappin Street, Sheffield, S1 3JD, United Kingdom, ^6^School of Engineering, Newcastle University, NE1 7RU, United Kingdom

*To whom correspondence should be addressed: Albers, S.-V., University of Freiburg, Institute for Biology II, Molecular Biology of Archaea, Schaenzlestrasse 1, 79104 Freiburg, Germany, phone: +49761/2032630, fax: +49761/2032773, email: [sonja.albers@biologie.uni-freiburg.de](mailto:sonja.albers@biologie.uni-freiburg.de); Lars-Oliver Essen, University of Marburg, Department of Chemistry, Hans-Meerwein-Strasse, 35032 Marburg, Germany, phone: +4964212822032, fax: +4964212822191, essen@chemie.uni-marburg.de

^#^These authors contributed equally

Contains: Figures S1-5, Tables S1-4

**
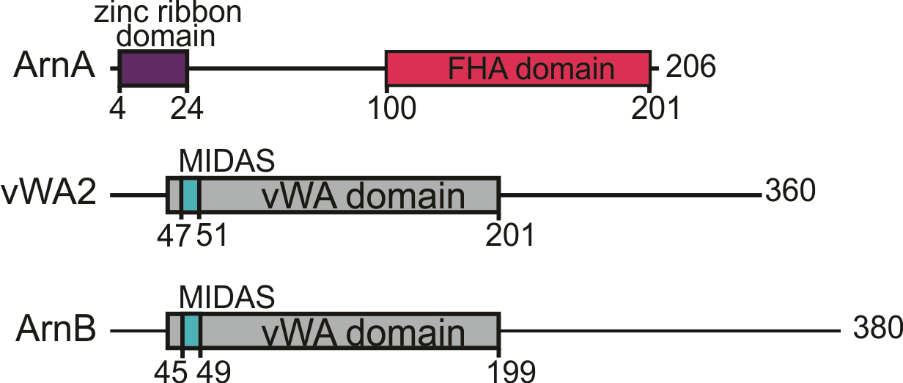
**

**Fig. S1.** Domain architectures of ArnA, vWA2 and ArnB.


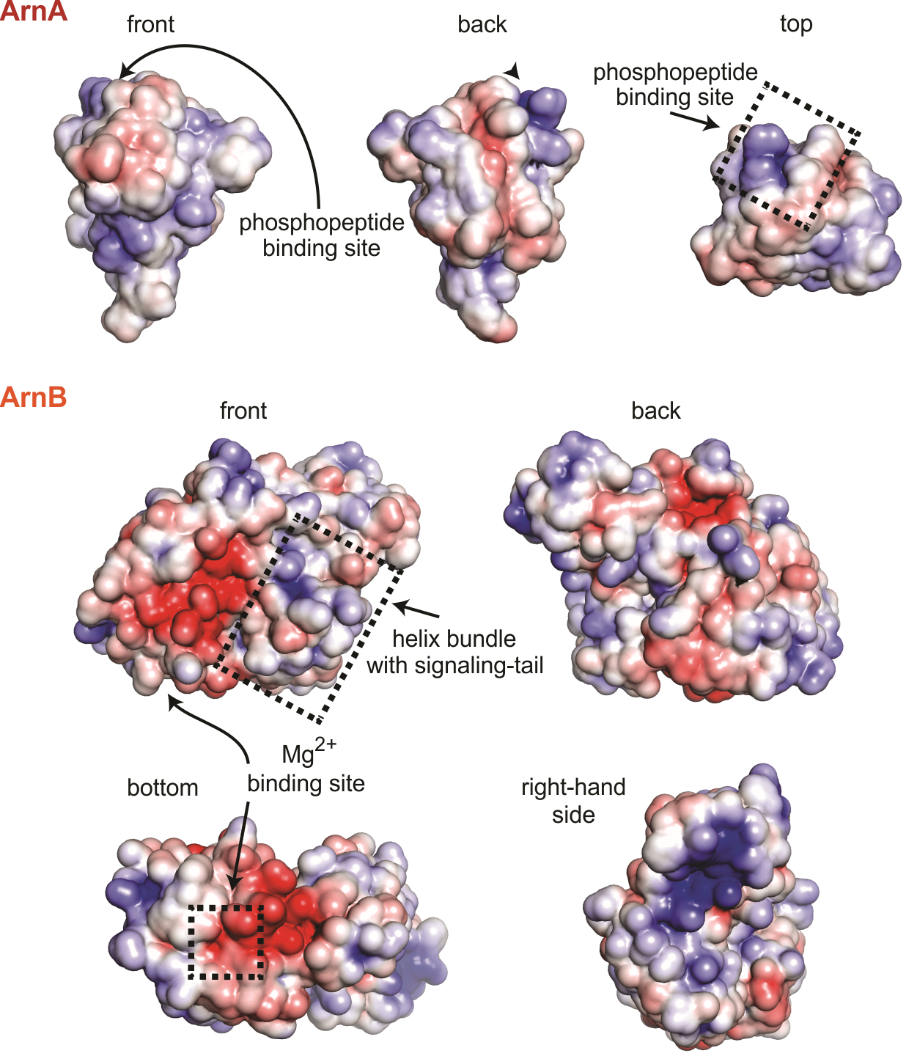

**Fig. S2.** Electrostatic surface potential of ArnA and the model of ArnB in different views on the proteins (± 5 kT/e; ion strength (+1/-1): 0.15, (-2/-2): 0; ion radius (+1/-1, +2/-2): 1.4). Negative and positive potentials on the surface are shown in red and blue, respectively. In ArnA and ArnB the phosphopeptide or Mg^2+^ binding site as well as the C-terminal helix bundle, respectively, are marked with arrows or a dotted box


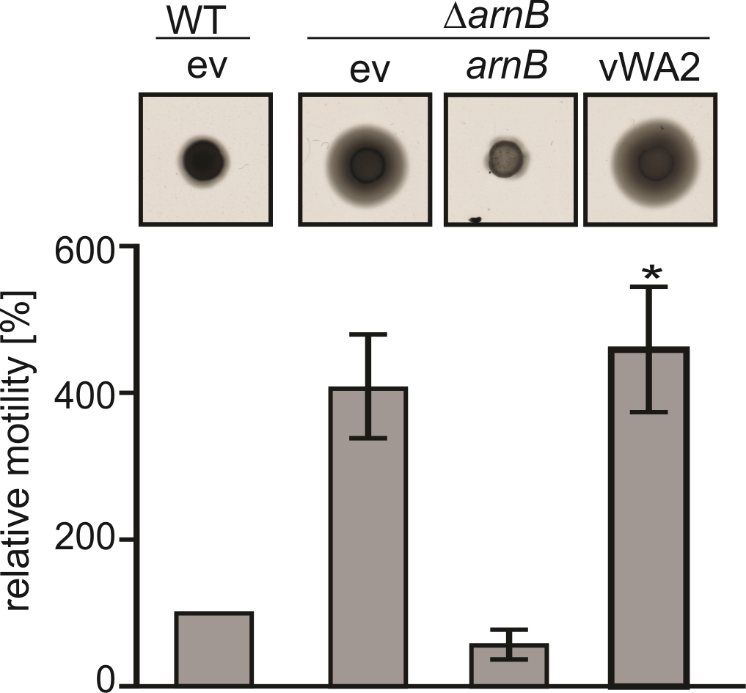

**Fig. S3.** Motility assay and swimming radius of *ΔarnB* complementation with vWA2 *in vivo*. *ΔarnB* strains were complemented with plasmid encoded *arnB* and *vWA2*, respectively and compared to WT and *ΔarnB* cells carrying the background vector (ev). All strains were analyzed for their motility phenotype on semi-solid plates. After growth for four days at 75 °C the swimming radii of the strains were calculated and compared to the WT strain carrying the background plasmid strain. Bars represent the mean relative swimming radius of three independent biological replicates with each six technical replicates normalized to the WT which was set to 100%. Statistical significance was analyzed using a student’s t-Test (unequal variance, two-tailed) compared to the WT strain. *P-*values < 0.05 are indicated by an asterisk.

**
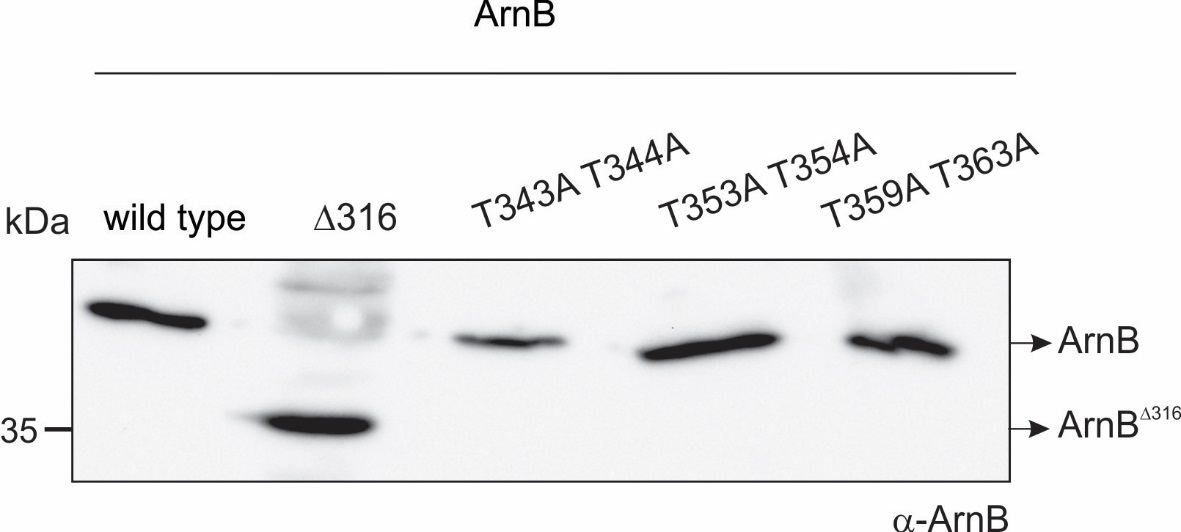

Fig. S4.** Plasmid-based expression of ArnB, ArnB^Δ316^, ArnB T343A T344A, ArnBT353A T354A, ArnBT359A T363A in the *S. acidocaldarius* Δ*arnB* strain. ArnB and mutant variants were expressed from a maltose inducible promoter. Cells were grown in maltose containing medium to early exponential phase (OD_600_ of 0.4) and harvested by centrifugation. The cell pellet was resuspended to a theoretical OD_600_ of 10 in PBS supplemented with SDS-loading dye. After incubation of the samples at 100°C for 10 min, samples were loaded on SDS-PAAGE, blotted and ArnB was detected using ArnB specific primary antibodies. Three independent experiments were performed and a representative Western-Blot is shown.


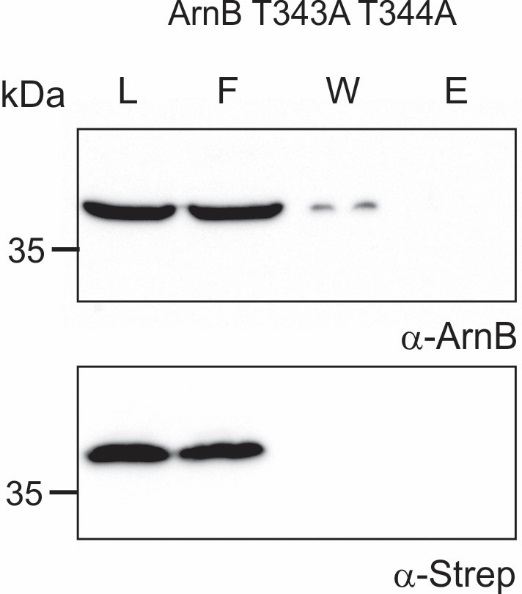

**Fig. S5.** ArnB T343A T344A does not bind to the streptactin coated magnetic beads. Load, Flow-through, wash and elution fractions obtained from pull-down analysis of cells grown in the presence of maltose to early exponential phase were analyzed by Western-Blot using either ArnB (upper) (in combination with goat-anti rabbit HRP coupled) or Strep-HRP (lower) antibodies. Three independent experiments were peformed and a representative Western-Blot is shown.

**Table S1.** Strains used in this study

| Strain | *Genotype* | Reference |
| --- | --- | --- |
| *E. coli* Top10 | *F- mcrA Δ(mrr-hsdRMS-mcrBC) φ80lacZΔM15 ΔlacX74 nupG recA1 araD139 Δ(ara-leu)7697 galE15 galK16 rpsL(Str^R^) endA1 λ^-^* | Invitrogen |
| *E. coli* BL21 (DE3) RIL | *B F- ompT hsdS(rB- mB-) dcm+ Tetr E. coli gal λ (DE3) endA Hte [argU ileY leuW Camr]* | Stratagene |
| *E. coli* Rosetta (DE3) pLysS | *F^-^ ompT hsdS_B_(R_B_^-^ m_B_^-^) gal dcm λ(DE3 [lacI lacUV5-T7 gene 1 ind1 sam7 nin5]) pLysSRARE (Cam^R^)* | Merck |
| *E. coli* ER1821 | *F- glnV44 e14-(McrA-) rfbD1 relA1 endA1 spoT1 thi-1*  *Δ(mcrC-mrr)114::IS10* | New England Biolabs |
| MW001 | *S. acidocaldarius DSM639 ΔpyrEF* | (1) |
| MW353 | *MW001 ΔarnB (saci1211)* | (2) |
| MW390 | *ΔarnB::arnB-*Strep . Based on MW353 as background strain. | This study |

| Table S2. Plasmids | |  |  |
| --- | --- | --- | --- |
| Plasmid | ***Purpose and cloning strategy*** | | **Reference** |
| pMZ1 | *E. coli* entry vector, C-terminal Strep- and His-tag, *ara* promoter | | (3) |
| pSA4 | *E.coli* expression vector. Derived from pET15b, contains multiple cloning site and N-terminal His_6_ tag of pSA5 | | (4) |
| pETDuet-1 | *E. coli* expression plasmid containing replicon CloE1 (pBR322) and two MCS, Amp^r^ | | Novagene |
| pSVA1450 | pCMalLacS-based vector for expression in *S. acidocaldarius, mal* promoter. Amp^r^ | | (5) |
| pSVA1009 | *arnC (saci1193)* with N-terminal His-tag cloned into MCSI of pETDuet-1 using *BclI/BamHI, PstI* | | (2) |
| pSVA1034 | *arnA (saci1210)* with C-terminal Strep-tag cloned into MCSII of pETDuet-1 with *NdeI, AvrII* | | (2) |
| pSVA1036 | *arnB (saci1211)* with C-terminal His-tag into MCSI of pETDuet-1 with *NcoI, BamHI* | | (2) |
| pSVA1068 | *vWA2 (saci1209)* with C-terminal His-tag cloned into pSA4 with *NcoI, BamHI* | | (2) |
| pSVA1076 | *arnD (saci1694)* with C-terminal His-tag cloned into MCSI of pETDuet-1 with *NcoI, PstI* | | (2) |
| pSVA1099 | For insertion of *arnB-strep (saci1211)* in genome. Used to generate MW390. Two fragments were generated from *S. acidocaldarius* MW001 genomic DNA using Primer 3117 + 3110 (*NcoI* site) and Primer 1582 + 3110 (*BamHI* site) harboring complementing overhangs. Fragments were fused via PCR using both fragments as templates and primers 3117 + 1582. The resulting fragment was subjected to restriction digest with *Nco*I and *BamHI* and ligated into pSVA406 (6) | | This study |
| pSVA2203 | Used for homologous overexpression of *vWA2-His-Strep* (*saci1209*). A PCR fragment covering the sequence of *saci1209* was amplified from genomic DNA of *S. acidocaldarius* MW001 using Primers 1642 (*NcoI*) and 1643 (*BamHI*). After restriction digest with *NcoI* and *BamHI* the fragment was ligated into pSVA1450. | | This study |
| pSVA2220 | *saci1211* cloned into pSVA1481 with *Nco*I, *Bam*HI | | (2) |
| pSVA2221 | *arnB (saci_1211)* with C-terminal His-Strep- *NcoI, EagI*. pSVA2220 (encodes *saci_1211)* was digested with *NcoI, EagI* and ligated with pSVA1450. | | This study |
| pSVA2270 | Used for cloning of pSVA2272 and pSVA2274. *arnBΔ316* (*saci_1211*) was amplified from *S. acidocaldarius* MW001 genomic DNA using primers 1597 (*Nco*I) + 3279 *(BamHI*). The resulting 953 bp fragment was subjected to restriction digest with *NcoI* and *BamHI* and ligated with PMZ1. | | This study |
| pSVA2272 | Used for heterologous expression of *arnB Δ316 (saci_1211*) in *E. coli*. pSVA2270 was subjected to restriction digest with *NcoI* and *BamHI* and the 953 bp fragment was ligated with pSA4. | | This study |
| pSVA2274 | Used for homologous expression of *arnB Δ316-His-Strep* (*saci_1211*). pSVA2270 (*arnB Δ316-His-Strep* in pMZ1) was subjected to restriction digest with *NcoI* and *EagI* and the resulting 1098 bp fragment was ligated in pSVA1450. | | This study |
| pSVA2277 | *arnB-T343A T344A (saci_1211)* with C-terminal His-Strep-tag cloned into pSVA1450. Generated by round PCR with primers 3273 and 3272. pSVA2221 was used as template. | | This study |
| pSVA2581 | *arnB-* T353A T354A *(saci_1211)* with C-terminal His-Strep-tag cloned into pSVA1450. Generated by round PCR with primers 9338 and 9339. pSVA2221 was used as template. | | This study |
| pSVA2582 | *arnB-* T359A T363A *(saci_1211)* with C-terminal His-Strep-tag cloned into pSVA1450. Generated by round PCR with primers 9340 and 9341. pSVA2221 was used as template | | This study |

**Table S3.** Primer

| Primer | 5’-3’ sequence | Purpose |
| --- | --- | --- |
| 1597 | GGGCCATGGGCTTACCCTAACTATCAAAC | Used Cloning of pSVA2270. |
| 3279 | CCCGGATCCGTTGGAGTTCAATTGATTAGC | Used for cloning of pSVA2270 |
| 3117 | GGGCCATGGACAAACAGTCCTGTATAG | Used for cloning of pSVA1099*.* |
| 3110 | CTCAAATTGTGGATGACTCCACGAACCTGATCCAGACCTCAACTTCTTAGTAACTTCAC | Used for cloning of pSVA1099. |
| 3111 | GGTTCGTGGAGTCATCCACAATTTGAGAAGTAAACAAGGACTAAATTTATCTTAGTTCTTCCTCAG | Used for cloning of pSVA1099. |
| 1582 | GGGGATCCGTAGACATTGAAGAAGGTAAAG | Used for cloning of pSVA1099*.* |
| 1642 | GGACCATGGCCACAGTTAGCGTTTCTTTAAAATC | Used for cloning of pSVA2203 |
| 1643 | GGCGGATCCTGTCCTCATTGTTTTTGTAACTTC | Used for cloning of pSVA2203, |
| 3272 | CTAATAGAGGCAGCTAGAAGAATAAGCGAAAGT | Used for cloning of pSVA2277 |
| 3273 | TATTCTTCTAGCTGCCTCTATTAGCTCCAT | Used for cloning of pSVA2277 |
| 9338 | TTCTTCTCGCTGCCTCTATACTTTCG | Used for cloning of pSVA2581 |
| 9339 | GTATAGAGGCAGCGAGAAGAATAGGTAC | Used for cloning of pSVA2581 |
| 9340 | CTTTCTCGCTTGCTCTACTGCACCTAT | Used for cloning of pSVA2581 |
| 9341 | TGCAGTAGAGCAAGCGAGAAAGATATC | Used for cloning of pSVA2581 |

**Table S4.** Crystallography data collection and refinement statistics.

|  | ArnA - Native | vWA2 - Native | vWA2 – wolframate soaking  Inflection | vWA2 – wolframate soaking  Peak | vWA2 – wolframate soaking  Remote |
| --- | --- | --- | --- | --- | --- |
| Data collection |  |  |  |  |  |
| Wavelength [Å] | 0.91801 | 0.70847 | 1.21522 | 1.21476 | 1.21120 |
| Space group | P4_1_ | C222_1_ | C222_1_ | C222_1_ | C222_1_ |
| Cell dimensions | *a*=43.6 Å, *b*=43.6 Å, *c*=71.8 Å, α=*β=γ=*90.0° | *a*=69.6 Å, *b*=74.3 Å, *c*=145.6 Å, α=*β=γ=*90.0° | *a*=69.7 Å, *b*=74.4 Å, *c*=145.5 Å, α=*β=γ=*90.0° | *a*=69.6 Å, *b*=74.3 Å, *c*=145.2 Å,  α=*β=γ=*90.0° | *a*=69.9 Å, *b*=74.7 Å, *c*=146.0 Å, α=*β=γ=*90.0° |
| Resolution [Å] | 30.81-1.75 (1.84-1.75) | 41.66-1.46 (1.54-1.46) | 48.48-2.00 (2.11-2.00) | 48.41-2.00 (2.11-2.00) | 35.23-2.00 (2.11-2.00) |
| Measured & unique reflections | 56429, 13490  (8149, 1923) | 352718, 65094 (51562, 9286) | 143178, 25412 (20619, 3634) | 142920, 25389 (20351, 3588) | 144971, 25779 (20753, 3671) |
| *R*_merge_ | 0.029 (0.520) | 0.047 (0.487) | 0.066 (0.412) | 0.060 (0.299) | 0.056 (0.356) |
| I/σ(I) | 32.1 (3.1) | 17.5 (3.5) | 17.3 (3.9) | 18.8 (5.2) | 18.7 (4.4) |
| Wilson *B* factor (Å^2^) | 24.8 | 17.4 | 24.7 | 22.7 | 26.7 |
| Mosaicity [°] | 0.156 | 0.118 | 0.139 | 0.141 | 0.147 |
| Completeness [%] | 99.6 (99.1) | 99.2 (97.7) | 98.5 (97.4) | 98.4 (96.6) | 98.5 (97.8) |
| Solvent content [%] | 51.5 | 45.5 | 45.5 | 45.4 | 46.2 |
| Multiplicity | 4.2 (4.2) | 5.4 (5.6) | 5.6 (5.7) | 5.6 (5.7) | 5.6 (5.7) |
| Refinement |  |  |  |  |  |
| Resolution [Å] | 30.81-1.75 | 41.66-1.46 |  |  |  |
| *R*_work_, *R*_free_ | 0.168, 0.189 | 0.165, 0.201 |  |  |  |
| Reflections (work, test) | 13488, 685 | 65082, 1340 |  |  |  |
| Residues | 116 | 365 |  |  |  |
| Water molecules | 120 | 323 |  |  |  |
| r.m.s.d. bonds [Å] | 0.005 | 0.011 |  |  |  |
| r.m.s.d. angles [°] | 1.002 | 1.340 |  |  |  |
| defined in mol. | P97 – F212 | A2 – G366 |  |  |  |

Values in parentheses denote the highest resolution shell.

**References**

1. Wagner, M., Berkner, S., Ajon, M., Driessen, A. J. M., Lipps, G., and Albers, S.-V. (2009) Expanding and understanding the genetic toolbox of the hyperthermophilic genus Sulfolobus. *Biochem. Soc. Trans.* **37**, 97–101

2. Reimann, J., Lassak, K., Khadouma, S., Ettema, T. J. G., Yang, N., Driessen, A. J. M., Klingl, A., and Albers, S.-V. (2012) Regulation of archaella expression by the FHA and von Willebrand domain-containing proteins ArnA and ArnB in Sulfolobus acidocaldarius. *Mol. Microbiol.* **86**, 24–36

3. Zolghadr, B., Weber, S., Szabó, Z., Driessen, a. J. M., and Albers, S. V. (2007) Identification of a system required for the functional surface localization of sugar binding proteins with class III signal peptides in Sulfolobus solfataricus. *Mol. Microbiol.* **64**, 795–806

4. Albers, S., Szabó, Z., and Arnold, J. M. (2003) Archaeal Homolog of Bacterial Type IV Prepilin Signal Peptidases with Broad Substrate Specificity. *J. Bacteriol.* **185**, 3918–3925

5. Wagner, M., Wagner, A., Ma, X., Kort, J. C., Ghosh, A., Rauch, B., Siebers, B., and Albers, S. V. (2014) Investigation of the malE promoter and MalR, a positive regulator of the maltose regulon, for an improved expression system in Sulfolobus acidocaldarius. *Appl. Environ. Microbiol.* **80**, 1072–1081

6. Wagner, M., van Wolferen, M., Wagner, A., Lassak, K., Meyer, B. H., Reimann, J., and Albers, S. V. (2012) Versatile genetic tool box for the crenarchaeote Sulfolobus acidocaldarius. *Front. Microbiol.* **3**, 1–12
